# Magnetic resonance imaging of pulse wave velocity in children aged 9 years to assess maternal influences on aortic stiffness in the offspring

**DOI:** 10.1186/1532-429X-14-S1-T8

**Published:** 2012-02-01

**Authors:** Jennifer A Bryant, Charles Peebles, Mark A Hanson, Sarah Crozier, Hazel Inskip, Sian Robinson, Philip C Calder, Cyrus Cooper, Keith Godfrey

**Affiliations:** 1 NIHR Biomedical Research Centre in Nutrition, Southampton, UK; 2 Cadiothoracic Radiology, Southampton University Hospitals NHS Trust, Southampton, UK; 3 Institute of Developmental Sciences, Southampton, UK; 4 MRC Lifecourse Epidemiology Unit, University of Southampton, Southampton, UK

## Background

Pulse wave velocity (PWV) is an indirect measure of vascular stiffness. Higher PWV is a recognised cardiovascular risk marker. Magnetic resonance imaging (MRI) is a non-invasive method of assessing PWV. Assessing maternal influences on offspring PWV is important as reduced fetal nutrient supply and impaired early development are linked with an increased risk of cardiovascular disease in adulthood. In multiparous women, changes in the uterine spiral arteries arising during previous pregnancies result in increased fetal nutrient supply. Rat studies have shown that changes in maternal fatty acid intake in pregnancy are associated with increased offspring arterial stiffness. Some studies of human adults suggest omega-3 fish oils reduce arterial stiffness. The objective of the study was to measure vascular stiffness using MRI, and examine maternal influences on vascular structure in children aged 9 years.

## Methods

Aortic PWV was assessed in 125 children aged 9 years (70 male, 55 female) using velocity-encoded MRI as part of a study of developmental influences on cardiovascular structure and function. Maternal diet had been assessed in early and late pregnancy, and the child's diet at age 9 years, using administered food frequency questionnaires.

## Results

PWV values fell within previously reported childhood ranges. Childhood aortic PWV was lower in offspring of multiparous mothers (3.32 m/sec, vs 3.45 m/sec in offspring of primiparous mothers, p=0.05). Higher maternal oily fish intake in pregnancy was associated with lower childhood aortic PWV (early pregnancy oily fish r=-0.19, p=0.047, n=106, late pregnancy oily fish r=-0.25, p=0.005, n=125, adjusting for child’s sex), but there was no association with the child’s current fish intake.

## Conclusions

MRI measurements of childhood aortic PWV indicate that the mother’s parity, and normal variations in maternal oily fish intake in pregnancy, may alter vascular development in utero - changing arterial structure and function with long-term consequences for cardiovascular risk in later life.

## Funding

This work was supported by funding from the British Heart Foundation and the National Institute for Health Research (Southampton NIHR Nutrition, Diet & Lifestyle Biomedical Research Unit).

